# Impact of Poor Sleep Quality on Task Switching and Reconfiguration Process Among University Students

**DOI:** 10.3390/bs15081054

**Published:** 2025-08-04

**Authors:** Shaoyang Ma, Yue Sun, Yunxin Jia, Jinfu Shi, Yekun Sun

**Affiliations:** 1 College of Psychology, North China University of Science and Technology, Tangshan 063210, China; sunyue@stu.ncst.edu.cn (Y.S.); jiayunxin@stu.ncst.edu.cn (Y.J.); shijinfu@ncst.edu.cn (J.S.); yekunsun@ncst.edu.cn (Y.S.); 2 Hebei Key Laboratory of Mental Health and Brain Science, Tangshan 063210, China; 3 School of Social and Political Sciences, University of Glasgow, Glasgow G12 8QQ, UK

**Keywords:** poor sleep quality, cognitive flexibility, task switching, task reconfiguration, university students

## Abstract

Task switching is an important cognitive function required for daily life, and task reconfiguration is one of the main explanations for the origins of switching costs. Studies have demonstrated that sleep significantly affects task switching abilities. However, there remains insufficient evidence on how poor sleep quality impacts task switching abilities among university students. A total of 85 university students were included in this study and classified into a poor sleep quality group (PSQ group, *n* = 47) and normal control group (NC group, *n* = 38) based on their Pittsburgh Sleep Quality Index scores. A task-cueing paradigm with different cue-to-target intervals (CTIs) was used to evaluate the participants’ task switching abilities and explore the process of task reconfiguration. An ANCOVA and subsequent simple effect analysis showed that the RT switching costs of the NC group decreased significantly as the CTI increased. However, there was no significant decrease in the PSQ group. Additionally, a significant difference was observed between different CTI conditions in repeat trials for the PSQ group, while no significant difference was observed for the NC group. The results showed that students with poor sleep quality exhibited slower task reconfiguration processes compared to the normal controls. Additionally, their capacity to resist interference and maintain task rules was found to be impaired.

## 1. Introduction

Cognitive flexibility enables rapid adaptation of thoughts and actions to dynamic external environments and internal states. Task switching, in which participants are required to perform a series of tasks, plays a pivotal role in assessing cognitive flexibility (Dajani & Uddin, 2015; Kiesel et al., 2010; Schmitz & Krämer, 2023). Sometimes the current task aligns with the rules of the preceding one, constituting a repeat task, while at other times the current task follows a different rule than the previous one, constituting a switch task. Compared to repeat tasks, switch tasks result in more errors and a longer reaction time (RT). This phenomenon is known as the switching cost (Rogers & Monsell, 1995; Schneider & Logan, 2007; G. Wylie & Allport, 2000), which serves as a crucial indicator for measuring task switching abilities (Fintor et al., 2019; Karayanidis et al., 2003; Nicholson et al., 2005). There are multiple theoretical perspectives regarding the source of switching costs, among which the task reconfiguration view suggests that switching costs reflect an endogenous, top-down, proactive control process (Monsell, 2003; Vandierendonck et al., 2010). Participants must engage in a task reconfiguration process for switch tasks contrasting with repeat tasks, and this additional process in switch tasks causes switching costs. Evidence supporting the reconfiguration view includes studies showing that extending the cue–target interval (CTI) to allow more time for task reconfiguration significantly reduces the switching costs (Meiran, 1996; Schmitter-Edgecombe & Langill, 2006). Specifically, a longer CTI leads to a decreased RT and increased accuracy for switch tasks, while the performance on repeat tasks remains unchanged. This perspective aligns with the preparatory effect theory, which suggests that allocating adequate preparatory time enhances the task switching performance (Schapkin et al., 2014; Wilckens et al., 2017).

Sleep is regarded as one of the most fundamental physiological processes in humans, playing a critical role in sustaining normal bodily and neurological functions. The prevalence of sleep disorders in the general population is around 30% (Fernandez-Mendoza & Vgontzas, 2013; Pavlova & Latreille, 2019). This issue may be particularly pronounced among university students, whose sleep disturbances predominantly manifest as an inadequate sleep duration and poor sleep quality (Becker et al., 2018; Galioto et al., 2015; L. Li et al., 2019). Poor sleep quality was associated with diminished cognitive performance and a faster decline in the processing speed among middle-aged and elderly people (Gobin et al., 2015; Köhler et al., 2023), as well as in healthy young adults (Benitez & Gunstad, 2012; Stiver et al., 2021). Evidence suggests that sleep can significantly affect various subcomponents of executive functions, including cognitive flexibility (Ballesio et al., 2019; Chen et al., 2016; Fortier-Brochu & Morin, 2014; Garcia et al., 2021; Sen & Tai, 2023).

Studies on sleep deprivation have revealed that inadequate sleep significantly affects cognitive flexibility and task switching abilities (Couyoumdjian et al., 2010; Honn et al., 2019; Slama et al., 2018; Stepan et al., 2022). On the contrary, a contrasting finding revealed that even with elevated levels of fatigue and drowsiness, sleep deprivation did not result in a significant increase in the switching costs (Nakashima et al., 2018). The disparities in the results may stem from variations in the experimental subjects and specific experimental paradigms. Furthermore, although sleep deprivation studies offer insights into the relationship between sleep and task switching to some extent, the research subjects predominantly consist of healthy individuals experiencing temporary sleep loss, differing from the population experiencing long-standing sleep quality problems. A study revealed that older adults with insomnia demonstrated a diminished preparatory effect on the accuracy of task switching compared to controls (Wilckens et al., 2017), implying that disrupted sleep may negatively affect the task reconfiguration process. However, there is limited evidence regarding the impact of sleep quality on the task switching capabilities of university students.

The objective of this study was to examine how poor sleep quality affects task switching among university students. University students were specifically targeted for three reasons: (1) Academic stress, irregular daily schedules, and lifestyle behaviors render university students especially vulnerable to poor sleep quality (Johansson et al., 2023; Schmickler et al., 2023; Shantakumari et al., 2024). (2) Late adolescence to early adulthood (typical university age) is a critical period for prefrontal cortex maturation, which directly supports task switching abilities (Weeda et al., 2014). (3) Early identification of the relationship between sleep and cognition in university students can facilitate timely interventions before chronic patterns develop, thereby reducing the impact of poor sleep quality on their academic performance and future employment prospects.

We utilized the Pittsburgh Sleep Quality Index (Buysse et al., 1989; Mollayeva et al., 2016) to screen university students for poor sleep quality and identify normal controls, employing a task-cueing paradigm with different CTIs to investigate the task reconfiguration process of the participants. We expected the following: (1) Due to the reconfiguration process, the RTs for switch trials in both groups were expected to decrease with an increasing CTI. In contrast, the RTs for repeat trials would show no significant differences between different CTI conditions. (2) Under both CTI conditions, the switching costs of students with poor sleep quality were expected to increase significantly. (3) In the long-CTI condition, students with poor sleep quality were expected to exhibit a smaller reduction in their RT for switch trials compared to normal controls, suggesting a slower reconfiguration process.

## 2. Materials and Methods

The ethical committee of the North China University of Science and Technology approved this study in accordance with the Declaration of Helsinki. Each participant provided written informed consent before participating in this study.

### 2.1. Participants

This study used convenience sampling.Ninety-two participants (aged 18–24) were recruited from the North China University of Science and Technology from 27 September 2023 to 7 June 2024. All the participants completed a demographic questionnaire, and they were right-handed with normal or corrected-to-normal vision. None of the participants had a history of significant neurological or psychiatric disorders, nor had they taken any psychotropic or anti-insomnia medications in the three months prior to the experiment. A series of neuropsychological measurements was used for participant screening. The sleep quality of the participants was measured using the Pittsburgh Sleep Quality Index (PSQI). Based on their PSQI scores, the participants were classified into a poor sleep quality group (PSQ group, PSQI scores > 5) and a normal control group (NC group, PSQI scores ≤ 5) (Backhaus et al., 2002). All the participants were screened for current depression (Beck Depression Inventory-13 scores > 7), intellectual disability (Wechsler Adult Intelligence Scale scores < 90), and daytime sleepiness symptoms (Epworth Sleepiness Scale score > 10, only applied to the NC group). Four participants (three with PSQ, one NC) were excluded from the data analysis due to a low accuracy rate (below 70%) in the task switching paradigm described below, and three normal controls were excluded due to their self-reported poor sleep the night before the experiment, which conflicted with their PSQI assessments. The demographic features and neuropsychological measures of the participants are given in Table 1.

### 2.2. Experimental Design and Procedure

**Figure 1:**
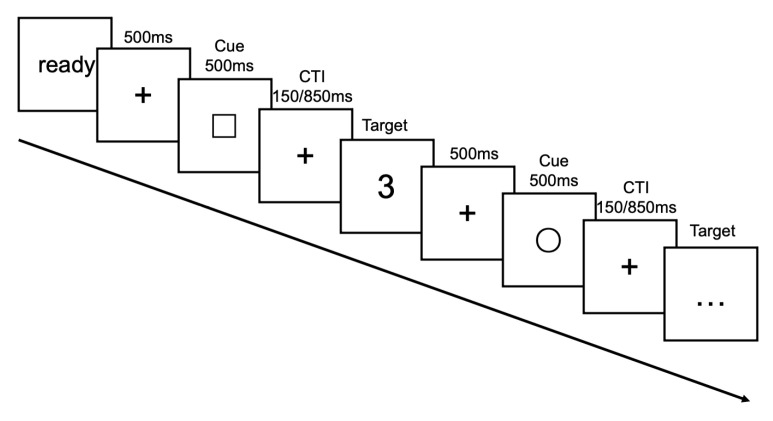
Representation of the procedure for the task-cueing paradigm.

Within two weeks of completing the neuropsychological measurements, a task-cueing paradigm was utilized to assess the participants’ task switching ability and cognitive flexibility. Figure 1 provides a schematic overview of the paradigm. At the beginning of each block, the term “Ready” appeared in the center of the screen, signaling participants to prepare for the forthcoming trial. Once ready, the participants initiated the experiment by pressing any key. Each trial commenced with a 500 ms fixation point, succeeded by a cue displayed on the screen for 500 ms, which could have been either a square or a circle. Following the cue, a cue-to-target interval (CTI: 150 ms versus 850 ms) ensued, after which a target stimulus, consisting of a digit ranging from 0 and 9, was presented. The participants were instructed to determine whether the digit was odd/even or larger than/less than 5, depending on whether the cue was a square or a circle, respectively. To indicate “odd” or “smaller than 5”, participants pressed the “F” key on the computer keyboard with their left index finger, while for “even” or “larger than 5”, they pressed the “J” key with their right index finger. The assignment of the response keys was counterbalanced across the participants within each group. A trial was coded as a repeat or switch trial based on whether the task cue was identical or different from that in the previous trial, respectively. Following the participant’s response (with a time limit of 4000 ms), a fixation point was displayed for 500 ms, succeeded by the subsequent trial. Prior to the formal experimentation phase, the participants underwent a thorough practice session to ensure their comprehension of and familiarity with the experimental protocol.

Preceding the task-cueing paradigm, the participants were asked to choose one of the following three options based on their sleep quality the night before: the same as usual, worse than usual, or better than usual. For participants in the NC group, those reporting ‘worse than usual’ would be excluded, while for participants in the PSQ group, those reporting ‘better than usual’ would be excluded. All the participants were confirmed to be healthy at the time of the experiment, with no active symptoms of influenza, COVID-19, or other acute illnesses.

The participants completed 4 blocks in the formal experiment, and self-paced breaks were available between the blocks. Each block consisted of 31 trials. Since the initial trial in each block was neither a repeat nor a switch trial, it was excluded from the data analysis. The paradigm employed a pseudo-randomized sequence to ensure that (a) the remaining 30 trials in each block had an equal number of repeat and switch trials; (b) across the 4 blocks, there were a total of 60 repeat trials and 60 switch trials, and within each type of trial, 150 ms CTIs and 850 ms CTIs were equally distributed; (c) there was no repetition of digits in any two consecutive trials; (d) there were no more than three repetitions of responses in consecutive trials. The measured variables of interest included the average RT and accuracy for both the switch and repeat trials.

### 2.3. Statistical Analysis

The data were processed using IBM SPSS Statistics 26.0. Inaccurate trials were excluded from both the calculation of the RT switching costs and the RT averages. No further data trimming was performed.

Independent-samples *t*-tests were conducted to assess the disparities in age and neuropsychological measurement outcomes. The homogeneity of the gender ratio between the groups was evaluated using Pearson’s chi-square test. The RT switching costs for each participant were calculated separately for the conditions of a short CTI (150 ms) and a long CTI (850 ms) as the mean switch RT minus the mean repeat RT.

To further explore the differences in the task performance across the task types, CTIs, and groups, separate repeated-measures ANCOVAs (Analyses of Covariance) were conducted for the percentage and mean RT of the correct trials, with the type (switch versus repeat) and CTI (short CTI versus long CTI) as within-subject factors, the group (PSQ versus NC) as a between-subject factor, and the Beck Depression Inventory-13 scores as a covariate. The identification of significant interactions in the repeated-measures ANCOVAs were followed with a simple effect analysis.

## 3. Results

The groups were matched in terms of participants’ gender ratio, age, and intelligence quotient. The PSQ group exhibited significantly higher PSQI and Epworth Sleepiness Scale scores compared to the NC group. Moreover, given the close relationship between poor sleep quality and moods, it is noteworthy that the PSQ group had significantly higher Beck Depression Inventory-13 scores than the NC group, despite neither group showing moderate-to-severe depressive symptoms. Table 1 presents the descriptive statistics.

### 3.1. Accuracy Rate

Table 2 presents the means and standard deviations of the accuracy rates for both groups across the different trial types and CTI conditions.

The ANCOVA revealed a significant main effect of the type (*F* (1,82) = 6.758, *p* = 0.01, *η*^2^ = 0.08). These findings suggest that repeat trials were associated with higher accuracy rates compared to switch trials. However, no significant main effects were found for either the CTI (*F* (1,82) = 0.36, *p* = 0.55, *η*^2^ < 0.01) or group (*F* (1,82) = 0.42, *p* = 0.52, *η*^2^ < 0.01). Furthermore, no significant interactions were observed between the group and type (*F* (1,82) = 0.82, *p* = 0.37, *η*^2^ = 0.01), the group and CTI (*F* (1,82) = 0.23, *p* = 0.64, *η*^2^ < 0.01), the type and CTI (*F* (1,82)= 0.69, *p* = 0.41, *η*^2^ < 0.01), or the group, CTI, and type (*F* (1,82) = 1.01, *p* = 0.32, *η*^2^ = 0.01). Additionally, no significant interactions were found between the covariate and the other factors. Detailed analysis results from the ANCOVA are presented in Table S1.

### 3.2. RT Switching Costs

The RT switching costs of both groups under different CTI conditions are presented in Table 2. The ANCOVA showed a marginally significant main effect of the CTI (*F* (1,82) = 0.85, *p* = 0.05, *η*^2^ = 0.05) and a significant interaction between the CTI and group (*F* (1,82) = 7.85, *p* < 0.01, *η*^2^ = 0.09). No significant interactions were found between the covariate and the other factors. Simple effect analyses with the covariate revealed a significant decrease in the RT switching costs as the CTI increased for the NC group (*F* (1,82) = 16.37, *p* < 0.001). However, in the PSQ group, no significant difference in the RT switching costs was observed between the two CTI conditions (*F* (1,82) = 0.10, *p* = 0.75). Additionally, in the short-CTI condition, there was no significant difference between the two groups’ RT switching costs (*F* (1,82)= 1.27, *p* = 0.26), but in the long-CTI condition, the RT switching costs of the PSQ group were higher than those of the NC group (*F* (1,82)= 5.91, *p* = 0.02). There was no significant main effect of the group (*F* (1,82)= 1.63, *p* =0.21, *η*^2^ = 0.02). Detailed analysis results from the ANCOVA are presented in Table S2, and the simple effect analysis results are presented in Table S3.

### 3.3. Reaction Time (RT)

**Figure 2:**
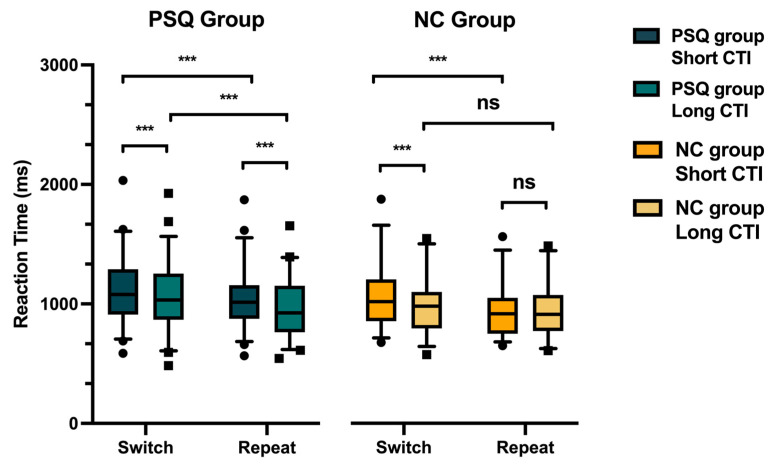
RT results. Boxplots depict RTs of switch trials in short-CTI condition, repeat trials in short-CTI condition, switch trials in long-CTI condition, and repeat trials in long-CTI condition for both groups. Whiskers span 5th–95th percentile range. *** *p* < 0.001; *ns* = not significant.

Figure 2 displays the RT for switch trials in the short-CTI condition, repeat trials in the short-CTI condition, switch trials in the long-CTI condition, and repeat trials in the long-CTI condition for both groups. The ANOVA revealed a significant main effect of the type (*F* (1,83) = 55.91, *p* < 0.001, *η*^2^ = 0.40) and CTI (*F* (1,83) = 32.66, *p* < 0.001, *η*^2^ = 0.28), along with a significant interaction among the group, type, and CTI (*F* (1,83) = 7.96, *p* < 0.01, *η*^2^ = 0.09).

The ANCOVA revealed a significant main effect of the type (*F* (1,82) = 47.49, *p* < 0.001, *η*^2^ = 0.37) and CTI (*F* (1,82) = 23.10, *p* < 0.001, *η*^2^ = 0.22), along with a significant interaction among the group, type, and CTI (*F* (1,82) = 8.06, *p* < 0.01, *η*^2^ = 0.09). There was no significant main effect of the group (*F* (1,82) = 1.20, *p* =0.28, *η*^2^ = 0.01), nor were there significant interactions among the covariate and other factors. Detailed analysis results from the ANCOVA are presented in Table S4.

Simple effect analyses of the covariate revealed significant differences in the RT between switch and repeat trials for both groups under the short-CTI condition (PSQ group: *F* (1,82)= 18.65, *p* < 0.001; NC group: *F* (1,82) = 21.76, *p* < 0.001). However, under the long-CTI condition, only the PSQ group showed a significant difference between the two types of trials (*F* (1,82) = 36.31, *p* < 0.001), while no significant difference was observed in the NC group (*F* (1,82) = 0.57, *p* = 0.45). Additionally, simple effect analyses showed significant differences between the short-CTI condition and the long-CTI condition in the switch trials for both the PSQ and NC groups (PSQ group: *F* (1,82) = 13.17, *p* < 0.001; NC group: *F* (1,82) = 22.91, *p* < 0.001). These findings indicate the presence of top-down task reconfiguration processes in both groups. However, in repeat trials, no significant difference in the RT between the two CTI conditions was observed for the NC group (*F* (1,82) = 0.08, *p* = 0.78), while a significant difference was observed in the PSQ group (*F* (1,82) = 12.99, *p* < 0.001). Detailed results from the simple effect analysis are presented in Table S5.

## 4. Discussion

The primary objective of the present study was to investigate the impact of poor sleep quality on task switching among university students.

Previous research on sleep-related task switching impairments has predominantly focused on elderly or clinical populations. In contrast, this study examined university students, demonstrating that even subclinical sleep deficits significantly impair the task reconfiguration efficiency during a critical neurodevelopmental period. Furthermore, unlike experimental sleep deprivation paradigms used with healthy adults, our investigation of naturally occurring poor sleep quality in non-clinical young adults exhibited higher ecological validity.

The results of the neuropsychological measurements showed that the PSQ group demonstrated higher levels of daytime sleepiness and increased depression compared to the NC group. Additionally, a substantial link exists between poor sleep quality and depression (Palmer & Alfano, 2017; Tsuno et al., 2005). This study excluded participants exhibiting moderate-to-severe depressive states. However, some individuals in the PSQ group exhibited mild depression (4 < Beck Depression Inventory-13 score ≤ 7), which resulted in a significant difference in the Beck Depression Inventory-13 scores between the groups. Prior studies examining the effects of depressive symptoms on executive functioning have primarily concentrated on major depressive disorder (Bortolato et al., 2014; Snyder, 2013; Stordal et al., 2004; Zheng et al., 2024). Although the impact of mild depression on executive function is less pronounced compared to that of severe depression, it remains a relevant factor that should not be disregarded (Grant et al., 2001; Paelecke-Habermann et al., 2005). Consequently, the Beck Depression Inventory-13 scores were included as a covariate in the group comparisons. The results indicated that the effect of sleep quality on task switching remained significant even after adjusting for depression, thereby supporting the role of sleep quality as an independent predictor, aligning with evidence that suggests that the influence of poor sleep quality on cognitive function operates independently of depression, particularly in young adults (Benitez & Gunstad, 2012).

The task-cueing paradigm employed in this study involved rule switching, which specifically activates endogenous reconfiguration processes driven by top-down cognitive control (Monsell, 2003). In contrast, sensory-modality switching paradigms primarily recruit bottom-up processes. Top-down reconfiguration requires working memory updating and inhibitory control and is predominantly mediated by the prefrontal and parietal cortex, regions particularly susceptible to sleep disorders (Killgore, 2010; Lanza et al., 2023).

The task switching results showed that the accuracy of both groups was higher in repeat trials compared to switch trials. However, there was no significant difference in accuracy between the groups. This lack of difference could potentially be attributed to the exclusion of participants with low accuracy rates (3 with PSQ and 1 NC), and for the remaining participants across both groups the task difficulty was comparable. Therefore, aligning with prior research (G. Wylie & Allport, 2000), this study focused on RT-related switching costs, which may be particularly prominent during the task reconfiguration process (Vandierendonck et al., 2010).

The RT switching costs were compared between the PSQ and NC groups under different CTI conditions. The results indicated that in both groups, the RT switching costs diminished as the CTI increased, implying the involvement of a task reconfiguration process (Schneider & Logan, 2007; G. Wylie & Allport, 2000). Additionally, as the CTI increased, the RT switching costs significantly decreased in the NC group, while the reduction in the PSQ group did not reach statistical significance. This indicates diminished preparatory effects and a slower reconfiguration process due to poor sleep quality. Unexpectedly, compared to the normal controls, university students with poor sleep quality did not exhibit a significant increase in the RT switching costs, as observed in sleep-deprived young individuals (Couyoumdjian et al., 2010; Honn et al., 2019; Slama et al., 2018; Stepan et al., 2022) and elderly individuals with insomnia (Wilckens et al., 2017).

To further investigate this, we compared the RT between the switch and repeat trials under different CTI conditions in the two groups. The results showed that under the short-CTI condition, both groups exhibited a significantly longer RT during switch trials compared to repeat trials. This suggested an ongoing process of task reconfiguration. However, under the long-CTI condition, the NC group did not exhibit a significant difference in the RT between the switch and repeat trials, whereas the PSQ group did. This indicates that as the CTI increased, the task reconfiguration process was gradually completed in the NC group, while it proceeded more slowly in the PSQ group. In other words, the preparatory effects in the PSQ group were diminished. Multiple studies have shown that preparation for task reconfiguration is associated with the activity of the frontal and parietal cortex. A cued task switching paradigm study and a pair-wise task switching paradigm study provided neurophysiological evidence that CNV-like negativities in the frontal and parietal regions are associated with switch-specific preparation in the reconfiguration process (Hsieh & Cheng, 2006; Poulsen et al., 2005). Complementing these event-related potential findings, time–frequency EEG analyses showed enhanced theta oscillations in midfrontal regions during preparation for switch trials compared to repeat trials (Cooper et al., 2019), suggesting oscillatory coordination of executive control. Additionally, fMRI evidence from a spatial Stroop task demonstrated that preparatory activation in the parietal cortex and dorsolateral prefrontal cortex enhances the cognitive processing efficiency (Stern et al., 2007). Based on the above evidence, the slower task reconfiguration process observed in university students with poor sleep quality in the present study may have been related to functional impairments in the frontal and parietal cortex caused by sleep disturbances. Future research could employ electrophysiological and neuroimaging techniques to further investigate the neural mechanisms underlying this phenomenon.

During repeat trials, the participants only needed to maintain the task rules without the need for task reconfiguration. The NC group did not show a significant difference in their response times between the two CTI conditions during repeat trials. However, the PSQ group exhibited significant longer response times under the short-CTI conditions during repeat trials compared to the long-CTI conditions, indicating a lower working memory capacity and ability to resist interference and maintain task rules with a shorter preparation time (Alsameen et al., 2021; Cho et al., 2015; Kiriş, 2022; J. Li et al., 2024; Xie et al., 2019). This result can also be explained by the increased mixed cost resulting from poor sleep quality. The mixing cost refers to the slower RT and decreased accuracy observed in repeat trials when they are mixed with switch trials within a block, compared to when all the repeat trials occur in a single-task block (Koch et al., 2005; Kray, 2006; Rubin & Meiran, 2005). The mixing cost reflects the working memory’s capacity to maintain and update information. Although working memory and cognitive flexibility are considered distinct subcomponents of executive function, there is a certain degree of interconnection between them. Evidence has suggested that the stronger the working memory capacity, the shorter the preparation time required for task switching (Schmitz & Krämer, 2023), aligning with the performance of the NC group in this study.

The PSQI has proven to be an effective and efficient tool for screening sleep quality perceptions in non-clinical populations, such as the student cohort in this study. Its ability to provide a comprehensive measure of sleep quality, incorporating both subjective evaluations and multiple domains of sleep disturbance, makes it a valuable instrument for identifying sleep-related issues within diverse groups. However, there are notable limitations to the PSQI, particularly with respect to the potential for bias in subjective self-reports. Although evidence suggests comparable validity of self-reported sleep measures between university students with and without depressive symptoms (Huang & Kämpfen, 2021), some studies indicate that a depressive mood can lead individuals to report poorer sleep quality and introduce subjective bias (Bliwise et al., 1993). In this study, participants in the PSQ group with elevated Beck Depression Inventory-13 scores may have overestimated their sleep disturbances, potentially confounding the results. While statistical adjustments for covariates can help mitigate this bias to some extent, the issue remains unresolved without the inclusion of objective measures of sleep. Future research should incorporate objective sleep measures, such as polysomnography, actigraphy, or other equipment to provide more accurate sleep assessments and reduce false positive associations caused by misclassification bias in subjective measures such as the sleep duration (Wang et al., 2025).

Three normal controls were excluded due to poor pre-experimental sleep that conflicted with their PSQI assessments. Evidence suggests that acute sleep loss resulting from single-night sleep disruption negatively impacts the next-day exercise performance (Craven et al., 2022) and working memory (Rångtell et al., 2019). Future research could further investigate the impact of acute sleep loss on task switching, thereby elucidating sleep’s critical role in cognitive functioning.

The sampling method used in this study also presents some concerns. A convenience sampling approach was employed by selecting university students from a single university in China, which limits the generalizability of the findings. First, the sample is not representative of other age groups, as the findings may not be applicable to children, older adults, or individuals in different life stages. Additionally, focusing on a single university in China introduces potential cultural and socio-economic biases, as the university student population may not reflect broader demographic groups in terms of their socio-economic status, educational environment, or lifestyle factors that could influence sleep and cognition. Consequently, the findings may not be applicable to students from different regions or countries with distinct cultural contexts, nor to non-student populations. To enhance the generalizability of future research, it would be beneficial to recruit more diverse samples in future studies, including individuals from various cultural, educational, and socio-economic backgrounds, and extend the study to clinical and community populations. Despite the sampling approach, the observed impact of sleep on cognitive function in these students remains clinically informative for campus mental health initiatives.

Although the participants were healthy and free from infection during the experimental period, the potential long-term effects of prior COVID-19 infection (long COVID) cannot be overlooked. Studies have shown that sleep disorders and cognitive impairment are common symptoms of long COVID and may persist for weeks to several months following recovery from the acute disease (Aiyegbusi et al., 2021; Cellai & O’kEefe, 2020; Davis et al., 2021). Furthermore, both the vaccination status and the type of vaccine received may influence subsequent sleep patterns and cognitive function in affected individuals (Gerhard et al., 2023; Percze et al., 2023). The absence of systematic screening for the participants’ COVID-19 infection status and vaccination history constitutes a limitation of this study. Consequently, potential confounding effects from prior COVID-19 infection or vaccine-related responses cannot be excluded. Future investigations that stratify participants by their infection status and vaccine platform would significantly strengthen the robustness and precision of these findings.

In addition to the direct symptoms caused by long COVID mentioned above, the COVID-19 pandemic indirectly affected university students’ mental health, sleep quality, and cognitive function through lifestyle changes such as social isolation, reduced levels of physical activity, and increased screen exposure time (Elsayed et al., 2023; Fila-Witecka et al., 2021; Zhang et al., 2022). Prior to the pandemic, the prevalence of poor sleep quality among university students was reported to exceed 50% (Becker et al., 2018; Lemma et al., 2012; Schlarb et al., 2017), and it may have worsened post-pandemic. Based on the negative impact of sleep quality on cognitive function found in this study, both universities and society should implement effective measures to improve students’ sleep quality. Future research should focus on exploring effective interventions for sleep disorders among university students.

Beyond the original findings and limitations noted above, the present study could be further improved in the following areas in future research. First, from certain perspectives it has been proposed that switching costs originate from exogenous and bottom-up reactive control processes (Houghton et al., 2009; Hübner et al., 2003; G. R. Wylie et al., 2004). This study specifically examined the influence of poor sleep quality on the endogenous task reconfiguration process, leaving room for further investigation into the effects of switching costs with alternative sources. Additionally, this study investigated the impacts of poor sleep quality on task switching behavior by comparing the RT and accuracy. However, it did not further explore the effects of poor sleep quality on neural activity during the task switching process.

## 5. Conclusions

Poor sleep quality can affect task reconfiguration during task switching. Students with poor sleep quality exhibited slower task reconfiguration and diminished preparatory effects compared to normal controls. Additionally, their capacity to resist interference and maintain task rules was found to be impaired.

## Figures and Tables

**Table 1:** Demographic features and neuropsychological measures (means, standard deviations, and group differences).

Features/Measures	PSQ Group (*n* = 47)	NC Group (*n* = 38)	*χ* ^2^ */t*	*p*
Male/Female	16:31	19:19	2.21	0.14
Age (Years)	19.51 (1.46)	19.45 (1.31)	0.21	0.84
Wechsler Adult Intelligence Scale	113.09 (8.84)	115.08 (9.49)	−1.00	0.32
Pittsburgh Sleep Index	8.72 (1.78)	2.37 (1.10)	20.18	<0.001
Epworth Sleepiness Scale	8.45 (5.97)	4.47 (3.05)	3.97	<0.001
Beck Depression Inventory	2.91 (2.62)	0.42 (0.89)	6.11	<0.001

Note: Pearson’s chi-square test was used to identify difference in gender ratio between PSQ group and NC group. Independent-sample *t*-tests were conducted to assess disparities in age and neuropsychological measurements.

**Table 2:** Accuracies and RT switching costs (means and standard deviations).

Statistical Outcomes from Group Comparisons	PSQ Group (*n* = 47)	NC Group (*n* = 38)
Accuracy with Short CTI in Switch Trials (%)	92.96 (6.23)	91.64 (6.94)
Accuracy with Short CTI in Repeat Trials (%)	93.36 (7.79)	94.11 (4.68)
Accuracy with Long CTI in Switch Trials (%)	93.22 (6.42)	92.54 (6.19)
Accuracy with Long CTI in Repeat Trials (%)	95.23 (5.96)	94.81 (4.66)
RT Switching Cost with Short CTI (ms)	90.40 (139.97)	124.49 (141.10)
RT Switching Cost with Long CTI (ms)	83.96 (119.86)	30.47 (82.77)

## Data Availability

The datasets used and analyzed during the current study are available from the corresponding author upon reasonable request.

## Supplementary Materials

The following supporting information can be downloaded at https://www.mdpi.com/article/10.3390/bs15081054/s1. Table S1: ANCOVA results regarding accuracy rates for both groups across different trial types and CTI conditions; Table S2: ANCOVA results regarding RT switching costs of both groups under different CTI conditions; Table S3: Simple effect analysis results regarding RT switching costs of both groups under different CTI conditions; Table S4: ANCOVA results regarding RT for both groups across different trial types and CTI conditions; Table S5: Simple effect analysis results regarding RT for both groups across different trial types and CTI conditions.

## Abbreviations

The following abbreviations are used in this manuscript:

PSQ	Poor sleep quality
NC	Normal control
CTI	Cue-to-target interval
RT	Reaction time
PSQI	Pittsburgh Sleep Quality Index
