# Supplementary material for: Impact of Poor Sleep Quality on Task Switching and Reconfiguration Process Among University Students
Source: Behav Sci (Basel). 2025 Aug 4;15(8):1054. doi: 10.3390/bs15081054 (PMC12383111; doi:10.3390/bs15081054)
Supplement: Supplementary file 1 [file behavsci-15-01054-s001.zip › behavsci-3671438-supplementary.pdf]

Supplementary Materials

Table S1. The ANCOVA results of accuracy rates for both groups across different trial types and CTI conditions.

**Effect**	**Factor**	***F***	***p***	η^2
Main Effect				
	Type	6.76	**0.01****	0.08
	CTI	0.36	0.56	<0.01
	Group	0.42	0.52	<0.01
	BDI	0.35	0.56	<0.01
Interaction Effect				
	Type * Group	0.82	0.37	0.01
	CTI * Group	0.23	0.64	<0.01
	Type * CTI	0.69	0.41	<0.01
	Type * CTI * Group	1.01	0.32	0.01
	Type * Beck Depression Inventory-13 scores	<0.01	0.97	<0.01
	CTI * Beck Depression Inventory-13 scores	1.83	0.18	0.02
	Type * CTI * Beck Depression Inventory-13 scores	0.20	0.66	<0.01

Note. **: significant.

Table S2. The ANCOVA results of RT switching costs of both groups under different CTI conditions

**Effect**	**Factor**	***F***	***p***	η^2
Main Effect				
	CTI	3.85	**0.05***	0.05
	Group	1.63	0.21	0.02
	BDI	2.94	0.09	0.04
Interaction Effect				
	CTI * Group	7.85	**<0.01*****	0.09
	CTI * Beck Depression Inventory-13 scores	0.65	0.42	<0.01

Note. *: marginally significant; ***: highly significant.

Table S3. The simple effect analyses results of RT switching costs of both groups under different CTI conditions

Effect Type	Factor	*F*	*p*
Within Group			
	PSQ Group: Long vs Short CTI	0.10	0.75
	NC Group: Long vs Short CTI	16.37	**<0.001******
Between Group			
	Short CTI: PSQ vs NC	1.27	0.26
	Long CTI: PSQ vs NC	5.91	**0.02*****

Note. **：significant; ***: highly significant.

Table S4. The ANCOVA results of RT for both groups across different trial types and CTI conditions.

Effect	Factor	*F*	*p*	η^2
Main Effect				
	Type	47.49	**<0.001*****	0.37
	CTI	23.10	**<0.001*****	0.22
	Group	1.20	0.28	0.01
	BDI	<0.01	0.95	<0.001
Interaction Effect				
	Type * Group	1.49	0.23	0.02
	CTI * Group	0.77	0.38	<0.01
	Type * CTI	3.68	0.06	0.04
	Type * CTI * Group	8.07	**<0.01*****	0.09
	Type * Beck Depression Inventory-13 scores	2.68	0.10	0.03
	CTI * Beck Depression Inventory-13 scores	0.42	0.52	<0.01
	Type * CTI * Beck Depression Inventory-13 scores	0.71	0.40	<0.01

Note. ***: highly significant.

Table S5. The simple effect analyses results of RT for both groups across different trial types and CTI conditions.

Effect Type	Factor	*F*	*p*
Within Group			
	PSQ Group in Short CTI: switch vs repeat	18.65	**<0.001*****
	PSQ Group in Long CTI: switch vs repeat	36.31	**<0.001*****
	NC Group in Short CTI: switch vs repeat	21.76	**<0.001*****
	NC Group in Long CTI: switch vs repeat	0.57	0.45
	PSQ Group in switch trials: Long vs Short CTI	13.17	**<0.001*****
	PSQ Group in repeat trials: Long vs Short CTI	12.99	**<0.001*****
	NC Group in switch trials: Long vs Short CTI	22.91	**<0.001*****
	NC Group in repeat trials: Long vs Short CTI	0.08	0.78
Between Group			
	Short CTI in switch trials: PSQ vs NC group	0.90	0.35
	Short CTI in repeat trials: PSQ vs NC group	2.14	0.15
	Long CTI in switch trials: PSQ vs NC group	2.25	0.14
	Long CTI in repeat trials: PSQ vs NC group	0.07	0.79

Note. ***: highly significant.
